# Dr. Landon B. Edwards

**Published:** 1911-01

**Authors:** 


Dr. Landon B. Edwards, of Richmond, Va., died at his home November 27, 1910, aged 65 years. He was educated at Randolph-Macon college in letters, served in the Confederate Army for two years, graduated in medicine from the Medical Department of the University of the city of New York in 1867, settled for practice at Lynchburg in 1868 where he married, moving to Richmond in 1872. He helped to organise the Medical Society of Virginia and was its secretary from the beginning. He established the *Virginia Medical Monthly* in 1874, which became the

*Virginia Medical Semi-Monthly* in 1896, and will be continued as such, by his son, Dr. Charles M. Edwards.

